# Supplementary material for: Cardiac-specific troponins in uncomplicated pregnancy and pre-eclampsia: A systematic review
Source: PLoS One. 2021 Feb 26;16(2):e0247946. doi: 10.1371/journal.pone.0247946 (PMC7909645; doi:10.1371/journal.pone.0247946)
Supplement: S2 Table — (DOCX) [file pone.0247946.s002.docx]

# S2 Table. Search strategy

| **Database** | **Search terms** | **Results*** |
| --- | --- | --- |
| Medline | \|  \| \| --- \| \| 1. Troponin I/ or troponin i.mp. \|  \| \| 2. limit 1 to (english language and humans) \|  \| \| 3. Troponin T/ or troponin t.mp. \|  \| \| 4. limit 3 to (english language and humans) \|  \| \| 5. Pregnancy/ or pregnancy.mp. \|  \| \| 6. limit 5 to (english language and humans) \|  \| \| 7. pregnancy complications.mp. or Pregnancy Complications/ \|  \| \| 8. limit 7 to (english language and humans) \|  \| \| 9. Obstetrics/ or obstetrics.mp. \|  \| \| 10. limit 9 to (english language and humans) \|  \| \| 11. 2 or 4 \|  \| \| 12. 6 or 8 or 10 \|  \| \| 13. 11 and 12 \|  \| | 209 |
| Embase | \| 1. troponin I/ or troponin i.mp. \|  \| \| --- \| --- \| \| 2. limit 1 to (human and english language) \|  \| \| 3. troponin T/ or troponin t.mp. \|  \| \| 4. limit 3 to (human and english language) \|  \| \| 5. pregnancy.mp. or pregnancy/ \|  \| \| 6. limit 5 to (human and english language) \|  \| \| 7. pregnancy complications.mp. or pregnancy complication/ \|  \| \| 8. limit 7 to (human and english language) \|  \| \| 9. obstetrics/ or obstetrics.mp. \|  \| \| 10. limit 9 to (human and english language) \|  \| \| 11. 2 or 4 \|  \| \| 12. 6 or 8 or 10 \|  \| \| 13. 11 and 12 \|  \| | 501 |

*Before removing duplicates
