# Supplementary material for: Cardiac-specific troponins in uncomplicated pregnancy and pre-eclampsia: A systematic review
Source: PLoS One. 2021 Feb 26;16(2):e0247946. doi: 10.1371/journal.pone.0247946 (PMC7909645; doi:10.1371/journal.pone.0247946)
Supplement: S3 Table — (DOCX) [file pone.0247946.s003.docx]

# S3 Table. Quality Assessment

| **Factor** | **Consideration** | **If yes: action** | **If no: action** |
| --- | --- | --- | --- |
| Bias | 1a) Was the population clearly defined? | None | Downgrade |
|  | 1b) Were appropriate exclusion criteria reported? | None | Downgrade |
|  | 1c) Were objective diagnostic criteria for pre-eclampsia and gestational hypertension defined? | None | Downgrade |
|  | 1d) Were demographic data for participants presented? | None | Downgrade |
|  | 1e) Was the distribution of cTn reported? | Upgrade | None |
| Imprecision | 2) Were standard deviations or appropriate confidence intervals reported for cTn? | None | Downgrade |
| Effects | 3) Was cTn and the degree of hypertension or severity of pre-eclampsia considered in a graded or dose-response approach? | Upgrade | None |

| **Study** | **Start quality** | **Positive factors** | **Negative factors** | **End quality** |
| --- | --- | --- | --- | --- |
| Adamcova, *et al.* | Low | 1a, 1d, 2 | 1b, 1e | Very low |
| Fleming, *et al.* |  | 1a-d | 1e, 2, 3 | Very low |
| Atalay, *et al.* |  | 1a-d, 2, 3 | 1e | Moderate |
| Aydin, *et al.* |  | 1a-d, 2 | 1e, 3 | Low |
| Rafik Hamad, *et al.* |  | 1a-d | 1e, 2, 3 | Very low |
| Pasupathi, *et al.* |  | 1a, 1c, 1d, 2 | 1b, 1e, 3 | Very low |
| Ersoy, *et al.* |  | 1a, 1b, 1d, 2 | - | Low |
| Ekun, *et al.* |  | 1a, 1b, | 1c-e, 2, 3 | Very low |
| Morton, *et al.* |  | 1a-d, 3 | 1e, 2 | Low |
| Ravichandran, *et al.* |  | 1a, 1b, 1d, 2, 3 | 1c, 1e | Low |
