# Supplementary material for: Cardiac-specific troponins in uncomplicated pregnancy and pre-eclampsia: A systematic review
Source: PLoS One. 2021 Feb 26;16(2):e0247946. doi: 10.1371/journal.pone.0247946 (PMC7909645; doi:10.1371/journal.pone.0247946)
Supplement: S4 Table — (DOCX) [file pone.0247946.s004.docx]

# S4 Table. Certainty of evidence

| **Outcome** | **Summary** | **Study design** | **Number of studies** | **Certainty of evidence** |
| --- | --- | --- | --- | --- |
| The association between levels of cTn and uncomplicated pregnancy | Nine studies reported levels of at least one cTn in women with uncomplicated pregnancies. Five of these studies reported reference intervals for the levels of cTn expected in healthy pregnant women.^a^ Four of these five studies of healthy pregnant women reported levels of cTn within the stated reference intervals, and one reported elevated levels about the upper reference limit. | All observational | 9 | Low |
| The association between levels of cTn and pre-eclampsia | Eight studies reported levels of at least one cTn in women with pre-eclampsia, although the definitions for pre-eclampsia varied. Five of these studies reported reference intervals for the levels of cTn expected in healthy pregnant women.^a^ Two studies reported elevated levels of cTn (above the reference interval) in women with pre-eclampsia and six studies reported elevated levels in women with pre-eclampsia compared to healthy pregnancy (not necessarily above the reference interval). One study reported low levels in all women but did not quantify these levels. | All observational | 8 | Low |

^a^The reference intervals were not stated to be pregnancy-specific, and were presumed to be extrapolated from studies of non-pregnant populations.
