# Supplementary material for: Cardiac-specific troponins in uncomplicated pregnancy and pre-eclampsia: A systematic review
Source: PLoS One. 2021 Feb 26;16(2):e0247946. doi: 10.1371/journal.pone.0247946 (PMC7909645; doi:10.1371/journal.pone.0247946)
Supplement: Attachment — Submitted filename: Response to reviewers.docx [file pone.0247946.s005.docx]

Response to reviewers

Dear Editor,

Thank you for your review and useful comments. We have addressed each in turn below. Please also find attached the updated manuscript, with and without tracked changes. We look forward to your decision.

Kind regards,

Dr Samuel Dockree

(on behalf of the authors)

Abstract
**10 studies only 8 summarised, what about others?**

*Rows 27-35:* We have re-worded this paragraph for clarity – it is now clear that all ten studies are accounted for (two groups of 7 studies are then referenced but these are not mutually exclusive).

**Is this sufficient evidence of cardiac stress or a leap?**

*Row 37:* This has been rephrased to describe the likely link to cardiac stress.

**Describe how to define 95% reference intervals specific to pregnancy, how do you know these are needed? Any studies examining difference between pregnant and non-pregnant controls?**

*Row 233:* We have added a paragraph in the discussion to address this, with an additional reference to a recent paper from our working group.

Introduction
**What is the biological basis and origin for circulating troponins? Please add this in brief.**

Sentence added with additional reference for pathophysiological mechanisms.
**Line 42. It would be better to state ‘used for’.**

*Row 45:* Changed

**Line 46. Can you state any details for the reference population that may relate to pregnancy e.g. is there a female-specific reference range, what are the characteristics (age, pre-post menopausal) of included women. What is the magnitude of difference between when and women in the 99th centile cut-off?**

*Row 53:* Paragraph inserted with the magnitude of sex-specific differences, with additional recent references, and a comment on demographics, including age.

**Line 59 Add ‘further elevated in pre-eclampsia’**

*Row 70:* Added

**Line 62 onwards. I would advise stating or very briefly summarising the basis for the techniques, given the significance of the differences you describe.**

*Row 75 onwards:* We have clarified the nature of the methodological advancements, which are driven by improving precision and allowing for earlier detection of the quantifiable circulating proteins.

Methods
**Line 93 Would non-pregnant female controls not have been a second relevant group for comparison?**

Thank you for raising this – we have also drawn reference to the female-specific non-pregnant reference intervals reported by Apple, *et al.* (2020), described in the background and discussion, although this was not the primary aim of the study.

**Line 97 Why exclude labour or immediate postpartum period as key risk times for cardiac events?**

*Row 264 onwards:* We have added a reference in the discussion (TOG 2013) in agreement with your comments about MI risk in the puerperium. However, we feel that the dramatic, multifactorial changes in labour would require a more detailed analysis than would be possible in this study of antenatal women, and would therefore be better addressed in a separate study.

**Were any limits based on study size applied and why not?**

*Row 100:* No – clarified

Results
**The large volume of search results indicate the search could potentially have been refined, however studies selected for full review all appears relevant to aim.**

**Line 142 correct to quantification?**

*Row 153:* Corrected

**153 This statement needs revision. I think this is insufficient evidence for a dose response to blood pressure. BP is a manifestation of pre-eclampsia that does not always reflect the severity of the disease, other factors would need to be controlled to begin to comment on this appropriately.**

Retracted

**Line 155 this is key information and should appear early in the results, please reposition. I think healthy non-pregnant women should have been included as an alternative comparator population. Can you confirm if they were included in the search (as you have identified this study).**

*Row 157 onwards:* Repositioned. Thank you for your comments regarding non-pregnant women – we refer to the results of the review by Apple, *et al.* which summarise the reference intervals reported in non-pregnant women, with which we draw comparisons.

**Line 156 please clarify, results were all within the normal range (<99th centile) advised by the manufacturer. Also specify that this reference interval was constructed using data from healthy male (or male plus female) non-pregnant individuals. This is also key information as you are justifying need for pregnancy-specific reference intervals.**

*Row 157 onwards:* Clarified as normal values – however the demographics (and percentile cut-off) for this reference population are not reported, further reiterating the importance of determining population-specific intervals.

**Line 160 onwards & Table 1. I would advise the statistical significance of these differences is specified in table 1.**

Added to both tables 1 and 2

Discussion (no line numbers to refer to in manuscript)

Line numbers added throughout

**‘TnI in women with an uncomplicated pregnancy was similar to non-pregnant women but has rarely been compared directly’. This is important and needs elaboration. Please include information from other authors here.**

*Row 219 onwards:* Paragraph inserted

**I completely agree with your decision not to pool and meta-analyse in the context of high heterogeneity, this is good practice.**

Conclusion
**‘There is an unmet clinical need for a suitably powered study to define the reference interval for cTn in uncomplicated pregnancy using highly sensitive methods’ A short practical description of this would be beneficial.**

*Row 233 onwards:* We agree, and have included a reference to a previous study from our working group, with suggested methods for this purpose.
